# Carbonic Anhydrase Interaction With Lipothioars Enites: A Novel Class of Isozymes I and II Inhibitors

**DOI:** 10.1155/MBD.1996.263

**Published:** 1996

**Authors:** Despina Timotheatou, Panayiotis V. Ioannou, Andrea Scozzafava, Fabrizio Briganti, Claudiu T. Supuran

**Affiliations:** 1 University of Patras Department of Chemistry Patras Greece; 2 Università degli Studi Laboratorio di Chimica Inorganica e Bioinorganica, Via Gino Capponi 7 Firenze I-50121 Italy

## Abstract

The interaction of carbonic anhydrase (CA) isozymes I and II with a series of As(III) derivatives, dialkyl and diaryl *rac*-2,3-dimyristoyloxypropyldithioarsonites, was investigated kinetically and spectrophotometrically, utilizing the native and Co(II)-substituted enzymes. Depending on the substitution pattern at the -As(SR)_2_ moiety of the investigated derivatives, inactive compounds were found for R = phenyl or naphthyl, and active ones for derivatives containing carboxyl groups (R = CH_2_COOH, cysteinyl and glutathionyl). Together with the arsonolipids previously investigated, the active compounds of this series - the "lipothioarsenites"- constitute a novel class of CA inhibitors that bind to the metal ion within the enzyme active site, as proved by changes in the electronic spectra of adducts of such inhibitors with Co(II)CA.


### CARBONIC ANHYDRASE INTERACTION WITH LIPOTHIOARSENITES: A NOVEL CLASS OF ISOZYMES I AND II INHIBITORS[#]

Despina Timotheatou[1], Panayiotis V. Ioannou[1], Andrea Scozzafava[2]

Fabrizio Briganti[2] and Claudiu T. Supuran*[2]

[1] University of Patras, Department of Chemistry, Patras, Greece
[2] Università degli Studi, Laboratorio di Chimica Inorganica e Bioinorganica,
Via Gino Capponi 7, I-50121, Firenze, Italiy

**Abstract**: The interaction of carbonic anhydrase (CA) isozymes I and II with a series of As(III) derivatives, dialkyl and diaryl *rac*-2,3-dimyristoyloxypropyldithioarsonites, was investigated kinetically and spectrophotometrically, utilizing the native and Co(II)-substituted enzymes. Depending on the substitution pattern at the -$As(SR)_2$ moiety of the investigated derivatives, inactive compounds were found for R = phenyl or naphthyl, and active ones for derivatives containing carboxyl groups (R = $CH_2COOH$, cysteinyl and glutathionyl). Together with the arsonolipids previously investigated, the active compounds of this series - the "lipothioarsenites"- constitute a novel class of CA inhibitors that bind to the metal ion within the enzyme active site, as proved by changes in the electronic spectra of adducts of such inhibitors with Co(II)CA.

### Introduction

Inhibition of the zinc enzyme carbonic anhydrase (CA, EC 4.2.1.1) with sulfonamide[2,3] or inorganic complexing anions[3,4] was extensively studied for at least two reasons: the first type of inhibitors led to the development of several valuable clinical drugs, used in the treatment or prevention of glaucoma,[5] gastric ulcers,[6] mountain sickness[7] or epilepsia[8] among others, whereas the second major class of inhibitors, the anions, allowed the discovery of mechanisms by which inhibition (and also catalysis) occur.[9-11] Besides these two classes of compounds, inhibition was also reported by aniline,[9] phenol,[12] thiophenols and heterocyclic mercaptans[13-15] as well as for some P(V)[16] and As(V) derivatives.[17]

Organic inhibitors from diverse classes possess a coordinating functional group (such as $SO_2NH_2$, SH, OH, $NH_2$, $AsO_3H_2$) generally attached to a bulky structural element (such as an aromatic/heterocyclic ring, possibly substituted with several side chains, but also aliphatic moieties are effective, for example perfluoroalkyl in the case of sulfonamides,[18] or dihydroxypropyl for arsonic acids[17]). Both these elements are extremely important for inhibition, as the first moiety generally interacts directly with the metal center or the solvent molecule bound to the metal ion,[2,3,9,19-22] whereas the second one assures stability to the enzyme-inhibitor adduct by means of cooperative interactions with amino acid side chains lining the active site. This was well illustrated in the case of the sulfonamide inhibitors, by the report of the X-ray crystallographic structures of several adducts of isozymes CA I - III with such inhibitors, by Liljas' group.[20,21]

Taking into account our interest in both sulfonamide as well as non-sulfonamide inhibitors of these enzymes[23,24] which might lead to the development of novel therapeutical approaches (such as the design of specific NMR imaging contrast agents[25], specific antiulcer drugs,[26] etc) we recently investigated a large series of compounds for their effect upon these enzymes. Interesting activity was discovered for some organo-element derivatives containing Ge(IV),[26] P(V)[16,27] or As(V)[17]. Here we report the extension of such studies on As(III) derivatives, more precisely, the interaction of thioarsenites with isozymes I and II of CA.

Mention should be made that thioarsenites of the trype $RAs(SR')_2$ were of pharmacological interest as antiparasite agents,[28a] since the nature of the R' moieties (present in the thiol from which they were prepared) could impart favorable solubility characteristics to them as well as to their metabolites, the biologically active arsenoso compounds ("RAsO").[28b]

[#] See ref.[1]

### Materials and Methods

Melting points were determined with a heating plate microscope and are not corrected; IR spectra were obtained in KBr pellets with a Perkin-Elmer 16PC FTIR spectrometer, whereas $^1$H-NMR spectra with a Varian T-60 or Bruker CPX200 apparatus in solvents specified in each case. Chemical shifts are expressed as $\delta$ values relative to $Me_4Si$ as internal standard. Some signals in the NMR spectra appear as broad singlets probably due to aggregation of the long chain lipid-like derivatives **1**. Optical rotations were measured on a Schmidt & Haensch Polatronic universal polarimeter using a 5 cm cell. Thin layer chromatography (tlc) was done using silicagel H from Merck. Visualisation was effected by spraying the microslides with 35% $H_2SO_4$ and charring. Elemental analysis were done by microcombustion with a Carlo Erba automated analyzer.

The As(III) derivatives **1** were prepared from *rac*-2,3-dimyristoyloxypropyl-arsonic acid and the corresponding thiols in acetone or DMSO as solvents, as described below. Buffers, 4-nitrophenyl acetate, acetonitrile, cysteine hydrochloride, mercaptoacetic acid, glutathione and inorganic reagents were from Sigma or Aldrich; 2-naphthalenethiol was from Merck and were used without additional purification.

Human CA I and CA II cDNAs were expressed in lon *Escherichia coli* strain SG20043 from the plasmids described by Forsman et al.[29] (the two plasmids were a gift from Prof. Sven Lindskog, Umea University, Sweden). Cell growth conditions were those described by Lindskog's group,[30] and enzymes were purified by affinity chromatography according to the method of Khalifah et al.[31] Enzyme concentrations were determined spectrophotometrically at 280 nm, utilizing a molar absorptivity of 49 $mM^{-1}.cm^{-1}$ for CA I and 54 $mM^{-1}.cm^{-1}$ for CA II, respectively, based on $M_r$ = 28.85 kDa for CA I, and 29.3 kDa for CA II, respectively.[32, 33] Apoenzymes were prepared by dialysing the zinc enzymes against 50 mM pyridine-2,6-dicarboxylic acid (Sigma) in 0.2 M phosphate buffer at 4°C for 2 hours.[34] The chelating agent was removed by dialysis against 20 mM Tris-$H_2SO_4$ (pH 7.5) and then 1.1 equivalents of $CoSO_4$ were added to the apoenzymes in order to obtain Co(II)CAs.

Electronic spectra were recorded with a Cary 3 spectrophotometer interfaced with an IBM PC. Initial rates of 4-nitrophenyl acetate hydrolysis were monitored spectrophotometrically, at 400 nm and 25°C, with a Cary 3 apparatus interfaced with an IBM compatible PC. Solutions of substrate were prepared in anhydrous acetonitrile; the substrate concentrations varied between $10^{-2}$ and $10^{-4}$ M. A molar absorption coefficient $\varepsilon$ = 18400 $M^{-1}.cm^{-1}$ was used for the 4-nitrophenolate formed by hydrolysis, in the conditions of the experiments (pH 7.80), as reported by Pocker and Stone.[35] Non-enzymatic hydrolysis rates were always subtracted from the observed rates. Stock solutions of inhibitors (10 mM) were prepared in DMSO and dilutions up to 0.1 $\mu$M were done with distilled deionized water. Duplicate experiments were done for each inhibitor, and the values reported throughout the paper are the averages of such results. $IC_{50}$ values represent the molarity of inhibitor producing a 50% decrease of enzyme specific activity for the investigated reaction.

### General procedure for the preparation of compounds of type 1

*rac*-2,3-Dimyristoylarsonic acid and the corresponding mercaptoderivative (thiophenol, 2-naphthalenethiol, mercaptoacetic acid, cysteine and glutathione, respectively) in a molar ratio of 1:4, were stirred in acetone (for the first three mercaptoderivatives) or DMSO (for the last two) at 50-55°C for 20-150 min, and then at room temperature for an additional 1.5-2 hours. TLC showed the reaction to be completed after approximately 60 min. The solution of **1d** was left overnight at -20°C and the obtained precipitate was centrifuged. When no precipitation occurred (**1e**), the acetone was evaporated *in vacuo* and the obtained solid recrystallized from petroleum ether. Compound **1a** was precipitated by adding water to its concentrated acetone solution. When the reactions were carried out in DMSO, the desired product was precipitated by addition of chloroform and petroleum ether (for **1b**) or water (for **1c**). Yields were in the range of 54-96%.

*Dicarboxymethyl rac-2,3-dimyristoyloxypropyldithioarsonite* **1a**: white amorphous solid, m.p. 33 - 35 ° C (from acetone); yield 96 %. IR (KBr), $cm^{-1}$: 3418 br (w); 2918 (s); 2850 (s); 1743 (vs); 1707 (vs); 1470 (m); 1304 (m); 1204 (s); 1164 (s). $^1$H-NMR ($CCl_4$), $\delta$, ppm: 0.90 (br s,6H, 2Me); 1.10-1.28 (m, 44H, $22CH_2$); 2.20 (br s, 6H, $CH_2As$ + $2CH_2COO$ from myristoyl); 3.60 (s,4H, $2SCH_2$); 4.20 (m,2H, $COOCH_2$); 5.40 (m,1H, COOCH). $R_f$ 0.95 in $CHCl_3$/AcOH 10:1, v/v. Analysis, found: C, 55.5; H, 8.7; S, 8.4 %; $C_{35}H_{65}O_8AsS_2$ requires: C, 55.8; H, 8.6; S, 8.5%.

*Dicysteinyl-rac-2,3-dimyristoyloxypropyldithioarsonite dihydrochloride* **1b**: white solid, m.p. 212°C (dec.); yield 73%. IR (KBr), $cm^{-1}$: 2922 (vs); 2852 (vs); 1742 (s); 1584 (vs); 1490 (vs); 1410 (s); 1340 (s); 1298 (s); 848 (s); 540 (s). $^1$H-NMR (DMSO-$d_6$), $\delta$, ppm: 0.95 (br s,6H, 2Me); 1.15-1.29 (m, 44H, $22CH_2$); 2.20 (br s, 6H, $CH_2As$ + $2CH_2COO$ from myristoyl); 3.72 (s,4H, $2SCH_2$); 3.97 (m, 2H, 2 CH from the cysteinyl moieties); 4.15 (m,2H, $COOCH_2$); 5.42 (m,1H, COOCH from the myristoyloxypropyl moiety). $R_f$ 0.62 in $CHCl_3$/MeOH/concentrated $NH_3$ 4:2:1, v/v. Analysis, found: C, 50.0; H, 8.3; N, 2.8; S, 7.3 %; $C_{37}H_{71}N_2O_8AsS_2$ 2HCl requires: C, 50.2; H, 8.2; N, 3.1; S, 7.2%.

*Diglutathionyl rac-2,3-dimyristoyloxypropyldithioarsonite* **1c**: white solid, m.p. 180 °C (dec.); yield 91%. IR (KBr), $cm^{-1}$: 3271 br (m); 2922 (s); 2852 (m); 1740 (m); 1650 (m); 1536 (m); 1414 (w); 1230 br (w). $^1H$-NMR (DMSO-$d_6$), δ, ppm: 0.95 (br s,6H, 2Me); 1.16-1.28 (m, 44H, $22CH_2$); 2.24 (br s, 6H, $CH_2As$ + $2CH_2COO$ from myristoyl); 2.56 (m, 4H, $2COCH_2$ from the glutamoyl moiety of glutatione); 2.78 (s, 4H, $2CH_2COO$ from the glycyl moiety of glutathione); 3.78 (s,4H, $2SCH_2$); 3.83 (m, 2H, 2CHNHCO from the cysteinyl moiety of glutathione) 3.92 (m, 2H, 2 CH from the glutamoyl moiety of glutatione); 4.14 (m,2H, $COOCH_2$); 5.44 (m,1H, COOCH from the myristoyloxypropyl moiety). $R_f$ 0.00 in $CHCl_3$/AcOH 10:1, v/v; $[\alpha]_D$ -13.3° (in chloroform, c 0.15). Analysis, found: C, 50.8; H, 7.3; N, 7.0; S, 5.3 %; $C_{49}H_{87}N_6O_{16}AsS_2$requires: C, 50.9; H, 7.5; N, 7.2; S, 5.5%.

*Diphenyl rac-2,3-dimyristoyloxypropyldithioarsonite* **1d**: white crystals, m.p. 35-36 °C (from acetonitrile); yield 66%. IR (KBr), $cm^{-1}$: 2916 (vs); 2848 (vs); 1736 (vs); 1726 (s); 1470 (s); 1180 (s); 1164 (s); 742 (s); 692 (s). $^1H$-NMR ($CCl_4$), δ, ppm: 0.90 (br s,6H, 2Me); 1.15-1.25 (m, 44H, $22CH_2$); 2.20 (br s, 6H, $CH_2As$ + $2CH_2COO$ from myristoyl); 4.10 (m,2H, $COOCH_2$); 5.30 (m,1H, COOCH); 7.15-7.60 (m,10H, 2Ph, ArH). $R_f$ 0.42 in $CHCl_3$/petroleum ether 1:1, v/v; $R_f$ 0.71 in $Et_2O$/petroleum ether 1:4, v/v. Analysis, found: C, 65.5; H, 8.7; S, 7.8 %; $C_{43}H_{69}O_4AsS_2$ requires: C, 65.4; H, 8.7; S, 8.1%.

*Di-(2-naphthyl) rac-2,3-dimyristoyloxypropyldithioarsonite* **1e**: white crystals, m.p. 132-135 °C (from petroleum ether); yield of 54%. IR (KBr), $cm^{-1}$: 2920 (m); 2850 (m); 1740 (m); 814 (s); 738 (s); 478 (s); $^1H$-NMR ($CCl_4$), δ, ppm: 0.90 (br s,6H, 2Me); 1.14-1.25 (m, 44H, $22CH_2$); 2.20 (br s, 6H, $CH_2As$ + $2CH_2COO$ from mirystoyl); 4.10 (m,2H, $COOCH_2$); 5.30 (m,1H, COOCH); 7.02-7.740 (m,14H, $2C_{10}H_7$, ArH). $R_f$ 0.47 in $CHCl_3$/petroleum ether 1:1, v/v; $R_f$ 0.55 in $Et_2O$/petroleum ether 1:4, v/v. Analysis, found: C, 68.5; H, 8.1; S, 7.3 %; $C_{51}H_{71}O_4AsS_2$ requires: C, 68.9; H, 8.2; S, 7.2%.

### Results and Discussion

Inhibition data with the newly synthesized racemic thioarsenites **1a-e** are shown in Table I, against the two red cell isozymes CA I and CA II.

As seen from data of Table I, the "lipothioarsenites" **1** (this name was coined due to the structural similarity of such derivatives to arsonolipids[36,37] **2**, for which CA II inhibitory properties were previously reported[17]) are able to inhibit both CA I and CA II only when in the $-As(SR)_2$ moiety there are COOH functionalities. Compounds substituted with aromatic groups (**1d,e**) are practically inactive (inhibition observed with these compounds at concentrations higher than 1 mM (data not shown) may be due to a non-specific hydrophobic interaction between the enzyme and the inhibitor).

For the three active derivatives, **1a-c**, the following observations can be made: (i) inhibitory power is greater when R moieties of the $-As(SR)_2$ group are smaller (with small differences of activity between the mercaptoacetic acid derivative **1a** and the cysteine derivative **1b**, but with a considerable loss of activity for the much bulkier glutathione derivative **1c**); (ii) derivatives **1a-c** act as better inhibitors of CA I compared to CA II, as observed for anion inhibitors but not for sulfonamides.[2,9,38] It is also obvious that in order for a lipothioarsenite to act as inhibitor, a coordinating group must be present in its molecule, which in this case is probably the carboxyl moiety. However, the acylated (myristoylated in our case) propanediol moiety of derivatives **1a-c** is also important for inhibition, since as seen from Table II, mercaptoacetic acid, *L*-cysteine or glutathione (the three thiols from which the thioarsenites were prepared) do not possess inhibitory effects at all.

Table I: CA I and II inhibition with arsenic derivatives 1 and 2 at 25°C. The concentration of 4-nitrophenyl acetate was $4.10^{-4}$ M (in acetonitrile), 10 mM Tris sulfate buffer, pH 7.0 ($\mu$ = 0.1 -$K_2SO_4$); [CA I] = 13.0 μM; [CA II] = 4.9 μM.

1

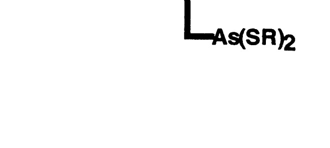

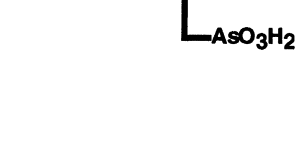

2

| | R | $IC_{50}$ (μM) CA I | CA II |
|---|---|---|---|
| **1a** | $CH_2COOH$ | 11 | 42 |
| **1b**[a] | $CH_2CH(NH_2)COOH$ | 15 | 49 |
| **1c**[b] | $CH_2CHNHCOCH_2CH(NH_2)COOH$ \ $CONHCH_2COOH$ | 152 | 230 |
| **1d** | Ph | >1000 | >1000 |
| **1e** | 2-naphthyl | >1000 | >1000 |
| **2** | $n$-$C_{13}H_{27}$ | 16 | 0.15[c] |

[a] Cysteine derivative; [b] Glutathione derivative; [c]From ref.[17].

Table II: CA I and II activation by thiols **3-5** in concentration of 10 μM. Other conditions were as described in Table I.

| Compound | % CA Activity [a] CA I | CA II |
|---|---|---|
| Mercaptoacetic acid, **3** | 113.4 | 115.3 |
| *L*-Cysteine, **4** | 122.3 | 121.5 |
| Glutathione, **5** | 139.7 | 142.5 |

[a] Enzyme activity in the absence of activator is taken as 100%.

Further information regarding the interaction of the active inhibitors with the enzyme was obtained by investigating the electronic spectrum of Co(II)-substituted CA II, and its adducts with inhibitors of type **1a-c**. It was proved earlier that inhibitors containing carboxylate moieties, such as formate,[39-41] acetate,[41] oxalate,[41] or benzoate[9] directly bind the metal ion in Co(II)CA. Co(II) is in pentaco-ordinated geometry in such adducts, with the three histidine residues of the enzyme, a water molecule and the carboxylate moiety of the inhibitor as ligands.[9,39-41] This has been recently confirmed by the report of the X-ray structure of the formate adduct of CA II. [42] Recently, other carboxylate inhibitors were reported, such as the non-steroidal antiinflammatory drugs ibuprofen **6** and flurbiprofen **7**,[43] or the positively-charged derivatives of *p*-aminosalicylic acid **8**.[44] It was proposed that such inhibitors directly bind to the metal ion through the negatively-charged oxygen atoms of the carboxylate moieties.[43,44]

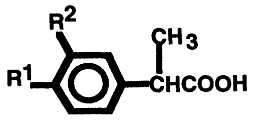

**6: R1=*i*-Bu; R2=H**
**7: R1=Ph; R2=F**

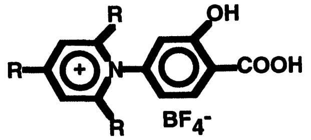

**8: R=alkyl or aryl**

In Table III electronic spectra of the adducts of derivatives **1a-d** as well as some carboxylate anions with Co(II)CA II are reported, which show that inhibitors **1a-c** interact with the metal ion by means of their $COO^-$ group(s), whereas the inactive derivative **1d** does not alter the spectrum of the cobalt-enzyme. The spectra of the three derivatives are quite similar with those of the benzoate or acetate adducts previously reported.[9,45,46,50] It should also be mentioned that adducts of Co(II)CA II with the thioarsenites **1** are not very stable. After 15 - 30 min these solutions showed the typical spectrum of Co(III)CA,[51] (data not shown) proving that oxidation of the metal ion occurred.

Table III: Electronic spectra of the adducts of Co(II)CA II with inhibitors **1** and carboxylate anions, at pH 7.5 and 0.2 mM enzyme concentration.

| Adduct | pH | Band position ,nm (molar absorptivity [$M^{-1}$ $cm^{-1}$]) |
|---|---|---|
| pure enzyme | 6.0 | 520 (180); 550 (250); 616.5 (135); 640 (100) |
| pure enzyme | 7.6 | 520 (240); 550 (310); 616.5 (220); 640 (200) |
| acetate | 7.6 | 472 (100); 515 sh (80); 555 (110); 709 sh (10) |
| benzoate | 7.6 | 480 sh (110); 507 sh (170); 555 (220); 591 sh (160) |
| **1a** | 7.6 | 475 (100); 510 sh (110); 555 (180); 596 sh (150) |
| **1b** | 7.6 | 477 (90); 510 sh (120); 555 (210); 595 sh (150) |
| **1c** | 7.6 | 482 (100); 507 sh (110); 555 (200); 600 sh (170) |
| **1d** | 7.6 | 519 (240); 550 (310); 616.5 (220); 640 (200) |

In conclusion, lipothioarsenites of type **1** possessing at least one COOH group act as inhibitors of isozyme CA I and II. They bind to the Co(II) ion in the cobalt-substituted enzyme in a manner similar to the carboxylate inhibitors, such as acetate or benzoate, the Co(II) having a trigonal-bipyramidal geometry.

**Acknowledgments**: This research was financed in part by the EU grant ERB CIPD CT 940051 associated to the MASIMO contract ERBCHRXCT 920072.

### References

1. This paper is Part 42 of the series Carbonic Anhydrase Inhibitors. Part 41: B.W.Clare and C.T.Supuran, *Eur.J.Med.Chem.*, in press.
2. T.H.Maren, *Pharmacol.Rev.*, **1967**, *47*, 595-782.
3. C.T.Supuran, "Carbonic anhydrase inhibitors", in "Carbonic Anhydrase and Modulation of Physiologic and Pathologic Processes in the Organism", I.Puscas Ed., Helicon, Timisoara **1994**, pp. 29-111.
4. C.T.Supuran, C.W.Conroy and T.H.Maren, *Proteins*, in press.
5. T.H.Maren, *J. Glaucoma*, **1995**, *4*, 49-62.
6. I.Puscas and C.T.Supuran, "Farmacologia clinica da ulcera peptica" in "Aparelho Digestivo", J.Coelho Ed., MEDSI, Rio de Janeiro, **1996**, pp. 1704-1734.
7. E.B.Larson, R.C.Roach, R.B.Schoene and T.F.Hornbein, *J.Am.Med.Ass.*, **1982**, *248*, 328-332.
8. D.M.Woodbury, "Carbonic anhydrase inhibitors" in "Antiepileptic Drugs: Mechanism of Action", G.H.Glaser, J.K.Penry and D.M.Woodbury Eds., Raven, New York, **1980**, pp. 617-633.
9. I.Bertini, G.Canti, C.Luchinat and A.Scozzafava, *J.Am.Chem.Soc.*, **1978**, *100*, 4873-4877.
10. S.Lindskog and A.Liljas, *Curr.Opin.Struct.Biol.*, **1993**, *3*, 915-920.
11. S.Lindskog and A.Liljas, *Roum.Chem.Quart.Rev.*, **1994**, *2*, 243-258.
12. I.Simonsson, B.H.Jonsson and S.Lindskog, *Biochem.Biophys.Res.Commun.*, **1982**, *108*, 1406-1412.
13. S.Schwimmer, *Enzymologia* **1969**, *37*, 163-173.
14. C.T.Supuran, M.D.Banciu, G.Botez and A.T.Balaban, *Rev.Roum.Chim.*, **1992**, *37*, 1375-1383.
15. C.T.Supuran, I.Saramet and M.D.Banciu, *Rev.Roum.Chim.*, **1995**, *40*, 1227-1232.
16. C.T.Supuran, V.Muresan, R.Popescu and I.Fenesan, *Main Group Met. Chem.*, **1995**, *18*, 629-632.
17. C.T.Supuran, S.V.Serves and P.V.Ioannou, *J.Inorg.Biochem.*, **1996**, *62*, 207-212.
18. T.H.Maren and C.W.Conroy, *J.Biol.Chem.*, **1993**, *268*, 26233-26239.
19. J.E.Coleman, *Ann.Rev.Pharmacol.*, **1975**, *15*, 221-243.
20. J.Vidgren, A.Svensson and A.Liljas, *Int.J.Biol.Macromol.*, **1993**, *15*, 97-100.
21. K.Hakansson and A.Liljas, *FEBS Lett.*, **1994**, *350*, 319-322.
22. A. Liljas, K.Hakansson, B.H.Jonsson and Y.Xue, *Eur.J.Biochem.*, **1994**, *219*, 1-10.
23. T.H.Maren, B.W.Clare and C.T.Supuran, *Roum Chem.Quart.Rev.*, **1994**, *2*, 259-282.
24. M.Coltau, C.T.Supuran and I.Puscas, "Arachidonic acid is an inhibitor of carbonic anhydrase I and the effect is reversed by indomethacin", in "Carbonic Anhydrase and Modulation of Physiologic and Pathologic Processes in the Organism", I.Puscas Ed., Helicon, Timisoara 1994, pp. 327-330.
25. I.Bertini, C.Luchinat, G.Parigi, A.Scozzafava and C.T.Supuran, *manuscript in preparation.*
26. I. Puscas, C.T.Supuran, M.Coltau, F.Chis and I.C.Puscas, *Rev.Roum.Biochim.*, **1994**, 31, 171-176.

27. A.Scozzafava, F.Briganti, C.T.Supuran, I.Fenesan, R.Popescu,V. Muresan and S.Farcas, *Main Group Met.Chem.*, in press.
28 a)H.Eagle and G.O.Doak, *Pharmacol.Rev.*, **1951**, *3*, 107-143; b) O.M. Ni Dhubhghaill and P.J.Sadler, *Struct.Bonding* **1991**, *78*, 129-190.
29. C.Forsman, G.Behravan, A.Osterman and B.H.Jonsson, *Acta Chem. Scand.*, **1988**, *B42*, 314-318.
30. G.Behravan, P.Jonasson, B.H.Jonsson and S.Lindskog, *Eur.J.Biochem.*, **1991**, *198*, 589-592.
31. R.G.Khalifah, D.J.Strader, S.H.Bryant and S.M.Gibson, *Biochemistry*, **1977**, *16*, 2241-2247.
32. P.O.Nyman and S.Lindskog, *Biochim.Biophys.Acta*, **1964**, *85*, 141-151.
33. L.E.Henderson, D.Henriksson and P.O.Nyman, *J.Biol.Chem.*, **1976**, *251*, 5457-5463.
34. J.B.Hunt, M.J.Rhee and C.B.Storm, *Anal.Biochem.*, **1977**, *79*, 614-617.
35. Y.Pocker and J.T.Stone, *Biochemistry*, **1967**, *6*, 668-679.
36. G.M.Tsivgoulis, D.N.Sotiropoulos and P.V.Ioannou, *Phosphorus, Sulfur, Silicon* **1991**, *57*, 189-197.
37. S.V.Serves, G.M.Tsivgoulis, D.N.Sotiropoulos, P.V.Ioannou and M.K.Jain, *Phosphorus, Sulfur, Silicon* **1992**, *71*, 99-105.
38. I. Bertini, C.Luchinat and A.Scozzafava, *Struct.Bonding*, **1982**, *48*, 45-82.
39. I.Bertini, C.Luchinat and A.Scozzafava, *Bioinorg.Chem.*, **1978**, *9*, 93-100.
40. I.Bertini, C.Luchinat and A.Scozzafava, *J.Chem.Soc.Dalton Trans.* **1977**, 1962-1965.
41. I.Bertini, C.Luchinat, R.Pierattelli and A.J.Vila, *Eur.J.Biochem.*, **1992**, *208*, 607-615.
42. K.Hakansson, M.Carlsson, A.Svensson and A.Liljas, *J.Mol.Biol.*, **1992**, *227*, 1192-1204.
43 I.M.Greene, M.Arifullah and A.D.Kenny, *Pharmacology*, **1992**, *45*, 278-284.
44. C.T.Supuran and M.D.Banciu, *Rev.Roum.Chim.*, **1995**, *40*, 891-900.
45. I.Bertini, C.Luchinat and A.Scozzafava, *Bioinorg.Chem.*, **1977**, 7, 225-231.
46. H.Shinar and G.Navon, *Eur.J.Biochem.*, **1979**, *93*, 313-317.

**Received: July 19, 1996 - Accepted: August 24, 1996 - Received in revised camera-ready format: October 4, 1996**

